# A Genome-Wide Study Replicates Linkage of 3p22-24 to Extreme Longevity in Humans and Identifies Possible Additional Loci

**DOI:** 10.1371/journal.pone.0034746

**Published:** 2012-04-10

**Authors:** Richard A. Kerber, Elizabeth O'Brien, Kenneth M. Boucher, Ken R. Smith, Richard M. Cawthon

**Affiliations:** 1 Department of Oncological Sciences, University of Utah, Salt Lake City, Utah, United States of America; 2 Huntsman Cancer Institute, University of Utah, Salt Lake City, Utah, United States of America; 3 Department of Human Genetics, University of Utah, Salt Lake City, Utah, United States of America; 4 Department of Family and Consumer Studies, Salt Lake City, Utah, United States of America; Ohio State University Medical Center, United States of America

## Abstract

**Background:**

Although there is abundant evidence that human longevity is heritable, efforts to map loci responsible for variation in human lifespan have had limited success.

**Methodology/Principal Findings:**

We identified individuals from a large multigenerational population database (the Utah Population Database) who exhibited high levels of both familial longevity and individual longevity. This selection identified 325 related “affected individuals”, defined as those in the top quartile for both *excess longevity* (*EL* = observed lifespan – expected lifespan) and *familial excess longevity* (*FEL* = weighted average *EL* across all relatives). A whole-genome scan for genetic linkage was performed on this sample using a panel of 1100 microsatellite markers. A strongly suggestive peak (Z = 4.2, Monte Carlo-adjusted p-value 0.09) was observed in the vicinity of D3S3547 on chromosome 3p24.1, at a point nearly identical to that reported recently by an independent team of researchers from Harvard Medical School (HMS) [1]. Meta-analysis of linkage scores on 3p from the two studies produced a minimum nominal p-value of 1.005×10^−9^ at 55 cM. Other potentially noteworthy peaks in our data occur on 18q23-24, 8q23, and 17q21. Meta-analysis results from combined UPDB and HMS data yielded additional support, but not formal replication, for linkage on 8q, 9q, and 17q.

**Conclusions/Significance:**

Corroboration of the linkage of exceptional longevity to 3p22-24 greatly strengthens the case that genes in this region affect variation in longevity and suggest, therefore, an important role in the regulation of human lifespan. Future efforts should include intensive study of the 3p22-24 region.

## Introduction

It has long been thought that related individuals share a familial predisposition to longevity, and for more than a century numerous studies have investigated the degree to which human longevity might be an inherited characteristic [2]–[12]. Most studies of this type have reported small (∼10%) to moderate (∼30%) heritability of human longevity, amid differences in definitions of longevity, methods of measuring it, ascertaining individuals who demonstrate it, and in various behavioral and environmental settings. These methodological differences likely account for much of the variation in the resulting estimates of the heritability of longevity.

To date the most intensive genome-wide scans for markers associated with human longevity have been based on data collected by the New England Centenarian Study (NECS). Puca et al. first reported results of a sib-pair linkage study using a whole-genome scan (308 subjects genotyped with 400 microsatellite markers) in 2001 [13]. Later Boyden and Kunkel [1] produced an updated sib pair linkage analysis using an expanded dataset (632 subjects, consisting of some of the NECS subjects and additional subjects recruited more recently) along with a high-density panel of 10,000 single nucleotide polymorphism (SNP) markers.

Lunetta and colleagues published a genome-wide association study (GWAS) of longevity among Framingham Study participants [14]. Despite numerous suggestive associations, Lunetta, et al. turned up no significant results based on their genome-wide SNP panel (of 100,000 markers). More recently, the Cohorts for Heart and Aging Research in Genomic Epidemiology (CHARGE) Consortium published a meta-analysis based on data from Framingham and three other cohort studies (the Age, Gene/Environment Susceptibility-Reykjavik Study, the Cardiovascular Health Study, and the Rotterdam Study); a confirmatory second stage genotyping included two additional datasets: the Leiden Longevity Study and the Danish 1905 cohort [15]. Combining these resources provided a substantially larger dataset; however, the meta-analysis over the 6 studies also failed to identify variants of genome-wide significance after adjusting for multiple comparisons. Nebel, et al. [16] conducted a GWAS for a sample of 763 Germans ranging in age from 94 to 110 years old, with a mean age 99.7 years; 1085 controls were drawn for the study, aged 45–77 years old, with a mean age of 60.2 years. After adjusting for 662,472 comparisons and post hoc quality control assessments, 16 SNPs were identified for confirmatory testing in an independent sample of 754 long-lived Germans and 860 controls. One SNP, rs4420638 near *APOC1*, exhibited a significant association with longevity both in the original and confirmatory tests. However, this SNP (rs4420638) is only 14 kb from the *APOE* locus; it is in strong linkage disequlibrium (LD) with the *APOE ε4* allele; and it has long been known that *APOE ε4* is associated with all-cause mortality [17]. Another recent GWAS study included 410 long-lived individuals and 553 younger controls from southern Italy [18]. In this study, the minor allele of one SNP (rs10491334) in the gene *CAMKIV*, was associated with reduced *CAMKIV* expression, and was underrepresented among long-lived individuals. *CAMKIV* appears to activate the proteins AKT, SIRT1, and FOX03A, all of which have been associated with increased longevity or longevity-associated mechanisms in humans and several model organisms [19]–[21].

GWAS studies of longevity must overcome several important challenges. The need to control for a very large number of comparisons compromises power considerably. The studies cited above are all quite small by current GWAS standards, and therefore, have only limited power to identify alleles associated with longevity. Selection of appropriate controls is a particular problem for studies of longevity. Controls are typically selected from the current population according to study design, and are by definition born one or more generations later than the long-lived individuals to which they are compared. Under such circumstances, selection, drift, and migration can affect allele frequencies in ways that might bias intergenerational GWAS results. Additional and potentially more substantial biases can result from behavioral changes across generations and time. Larger and more robust GWASs will undoubtedly contribute importantly to our understanding of the genetics of longevity; but for now, other approaches, including family studies, remain competitive.

Here we report the results of the Fertility, Longevity and Aging (FLAG) study, a genome-wide genetic linkage study of familial exceptional longevity. Subjects were selected from the Utah Population Database [22], [23] under a design that differs from most others in several important characteristics: 1) subjects were selected on the basis of both individual longevity, and a strong family history of longevity; 2) relatives of varying degrees of relationship were included; and 3) linkage analysis was based on a microsatellite marker set (deCODE Genetics 1100 marker set) more appropriate than a conventional 400 marker linkage panel to the extended family structures of subjects drawn from the UPDB, and hence, relatively short regions of identity by descent.

## Methods

### Utah Population Database

The Utah Population Database (UPDB) is a repository of longitudinal information on Utahans and their families. Originally constructed from genealogical data [23], the database has developed by successive record linking to integrate cancer registry data, Utah death certificates, U.S. Census data, Utah birth certificates, Utah driver license records, and more recently, medical records data. Currently the database includes information pertaining to approximately 7 million individuals, many of whom are integrated into multigenerational pedigree networks 2 to 14 generations deep [22].

### Subjects

In two previous studies, we examined the influence of family history on longevity in the UPDB: we defined *excess longevity* (*EL*) as the difference between observed and expected lifespan for an individual; and we defined *familial excess longevity* (*FEL*) as a kinship- weighted average of *EL* across all the family members of a subject [11], [12]. For this study, subjects were identified after evaluating several methods for selecting them, using simulated data and various combinations of *EL* and *FEL*. The goal was to maximize the positive predictive value (the probability that a subject carried a simulated longevity extending allele, given lower all-cause mortality) while maximizing potential sample size.

Simulations assumed a genetic variant with autosomal dominant inheritance, and an allele frequency of 0.01. Penetrance was expressed as an all-cause mortality hazard ratio, and set at 0.5. Although we considered multiple approaches to selecting subjects based on individual longevity and family longevity scores, including Markov chain Monte Carlo approaches, we found that simply combining top quartile values of both *EL* and *FEL* (*EL*≥3.0; *FEL*≥1.75) outperformed the other methods and was simpler to administer. Simulation tests under different assumptions about allele frequency and penetrance altered the sensitivity and positive predictive value of the selection criteria, but did not change the relative performance of the methods.

The University of Utah Health Sciences Institutional Review Board and the University of Louisville Biomedical Institutional Review Board approved the study protocol; all recruited subjects provided their written consent to be included in this study. A total of 732 study subjects were genotyped by deCODE Genetics, Reykjavik, Iceland. Of these, 433 were identified as expressing an excess longevity phenotype (ELP), and were considered “affected”. We recruited and genotyped 139 offspring of affected individuals, and considered these putative carriers of the ELP. Last, we recruited and genotyped 160 “controls”: 54 offspring of ***deceased siblings*** of “affected” individuals were considered putative non-carriers of the long-lived phenotype; and106 randomly drawn individuals from the UPDB population complete the control group. Ages of the 433 ELP affected individuals ranged from 86 to 109 years old. Only 325 of these were related to another genotyped affected individual. Subjects were enrolled between August 2003 and January 2009. By 2009, 117 ELP affected subjects had died. The total number of affected pairs over all classes of relatives was 607, because many affected individuals were related to multiple others through multiple lines of descent. The complex and overlapping nature of the genealogical data makes delineation of discrete “families” difficult: by the broadest definition, all study subjects could be connected to one another by one or more known genealogical links (including marriage). On the other hand, the vast majority of affected pairs had kinship values of zero, i.e. they were not biologically related by any known common ancestry. In spite of the genealogical complexity, no affected individual was measurably inbred.

Table 1 gives the demographic characteristics of study subjects. The unexpected relative excess of males is explained by two factors: first, the computation of excess longevity adjusts for sex, effectively counting males as females approximately two years older. Second, and more importantly, it proved easier to locate extremely aged men, by current residence, than women in Utah. Women in the UPDB are more likely to have changed their names as a result of marriage, are less likely to have driver's licenses (especially at advanced ages), and are less likely to have health care billing records in their own names than are men. All these characteristics make it more difficult to find a correct current residence using the available records.

**Table 1:** Demographic Characteristics of Study Subjects.

	Affected		Affected with Typed Relative		
Age	Male	Female	Male	Female	Total
86–89	36	5	27	5	32
90–92	65	60	51	49	100
93–95	65	53	50	41	91
96–97	43	21	31	16	47
98–99	19	18	7	13	20
100+	19	29	14	21	35
Total	247	186	180	145	325

Typed Affected Relative Pairs		
Degree	Total	Closest
1st (sibs)	63	95
2nd	2	4
3rd	32	27
4th	27	25
5th	77	47
6th	93	27
7th	107	31
8th or more	206	69
**Total**	607	325

Relative Pairs: “Total” is a count of all pairs of a given type among genotyped affected individuals. “Closest” is a tabulation of the closest genotyped affected relative among all genotyped affected individuals.

### Genotypes

Genotyping was performed at deCODE Genetics, Reykjavik, Iceland, using the deCODE 1100 microsatellite marker set. Here we report only the autosomal marker results. Any marker successfully typed in at least 50% of subjects was analyzed. The maximum number of markers successfully typed in any sample was 1074, and 1051 markers (97.8%) met the analysis inclusion criterion (returned types for >50% of subjects). The number of alleles observed per marker ranged from 2 (D9S1152, DG19S135) to 38 (DG19S265). Average marker spacing over the 22 autosomes was 3.4 cM. Allele frequencies were estimated from control subjects by simple counting, and checked against the HapMap European Reference (CEU) allele frequencies.

### Analytical methods

The relatively large and complex sample – in particular the large number of ungenotyped individuals intervening in the paths of relationship between genotyped affected family members – presented an analytical challenge. It proved impossible with existing software to obtain reliable estimates of identity by descent (IBD) probabilities. Exact estimation with software such as Genehunter [24], Allegro [25], or Merlin [26] required more computer memory than was available; Markov chain Monte Carlo (MCMC) methods, as employed by Loki [27], Genibd [28], SOLAR [29], and SIMWALK [30] failed to converge or converged to obviously incorrect solutions, after consuming literally weeks of CPU time.

Therefore, we used a simplified version of the venerable affected pedigree member (APM) test, originally devised by Weeks and Lange [31], as a primary test for linkage of EL to each chromosomal region. Let *G_i1_* and *G_i2_* represent the two alleles of a given marker carried by person *i*; *G_j1_* and *G_j2_* are the corresponding alleles carried by a family member *j* of person *i*, and *q(g)* is the allele frequency for marker allele *g*. Then the sharing statistic *S_ij_ is*:
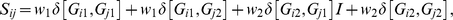


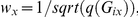
where δ is the Kronecker delta function (1 if the two arguments are equal, 0 otherwise).

The APM statistic is the sum of the random variable S*_ij_* over all *i* and *j*. As a test for significance, a null distribution of marker alleles was generated using a “gene-dropping” algorithm: first alleles are randomly assigned to founders in proportion to their expected frequency; then they are transmitted by descent throughout the pedigree. Five hundred iterations of this algorithm were performed for each marker, and Z-scores computed as the difference between the mean score under the null and the observed score, divided by the null standard deviation. P-values were estimated by reference to a standard normal distribution, and empirical p-values computed as the number of times a randomly-generated test statistic exceeded each observed value.

Weeks and Lange also describe a more powerful multipoint variation of the APM statistic [32]. The multipoint statistic is simply the sum of *S_ij_* for a set of adjacent markers, but the appropriate null distribution must then account for non-independence among markers. To accommodate this requirement, the gene-dropping algorithm (above) was modified so that “chromosomes” – sets of linked markers– passed from parents to offspring with between-marker recombination probabilities assigned by Haldane's mapping function [33]. We simulated 500 null values per chromosome. Z scores and p-values were computed as described above. Multipoint APM (MAPM) scores were also computed for all pairs of markers adjacent to a locus on either side, at 1 centimorgan (cM) intervals; the technique weights the contribution of each marker inversely by the relative probability of recombination with the adjoining locus.

To adjust for multiple comparisons, we refer to the distribution of simulated Z-scores across all markers and multipoint intervals, and compute the experiment-wise p-values as described by Churchill and Doerge [34].

For selected regions that showed evidence of linkage in both the HMS and FLAG data (3p and 18q), we generated meta-nominal p-values following the method of Dempfle and Loesgen [35]. These p-values were adjusted for multiple comparisons by reference to the quantiles of the simulated Z-score distribution described above.

## Results

**Figure 1:**
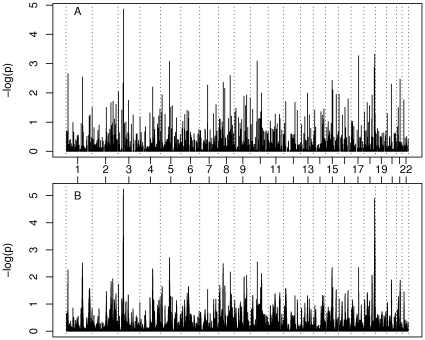
FLAG study linkage results for all autosomes. A) nominal singlepoint p-values; B) nominal multipoint p-values.

Figure 1 shows p-values for linkage across the genome from the APM scan. Table 2 lists 19 markers with unadjusted asymptotic p-values<0.01. Table 3 lists 17 multipoint regions with multiple comparison-adjusted p-values<1.0. The most noteworthy linkage scores indicate regions of interest on chromosomes 3p24 and 18q22. In each of these two regions, markers adjacent to the one giving the highest linkage signal, also show evidence of linkage. The strongest linkage signal was observed at D3S3547, on chromosome 3p24.1 @56.45 cM (or 30.1 Mb). D3S1266 and D3S3547 are immediately adjacent to one another on 3p24.1, as are D18S469 and D18S1161 on 18q22.3. Tables 2 and 3 also show p-values adjusted for multiple comparisons, as quantiles of the minimum simulated p-value observed at each locus. Alone, none of these results achieves genome-wide significance after adjusting for the number of hypotheses tested. The most significant genome-wide result is the singlepoint estimate for D3S3547 (adjusted p = 0.09). The same approximate location (@ 55 cM) yields a multipoint adjusted p of 0.20. The linked region on 18q22-23 appears more significant by multipoint (@109 cM; adjusted p = 0.25) than by the singlepoint (adjusted p = 0.59 for D18S1161 @111.05 cM) method.

**Table 2:** Markers linked to exceptional longevity with nominal p-value<0.01.

Chromosome	Marker	Map Position (cM)	Z	Asymptoticp-val	Adjusted p-value
1	D1S2667	20.36	2.84	0.00223	0.966
1	D1S2628	168.68	2.76	0.00288	0.976
3	D3S1297	5.05	2.35	0.00928	1.000
3	D3S1266	52.22	2.60	0.00469	0.998
3	D3S3547	56.45	4.19	0.00001	0.090
4	D4S1615	129.75	2.49	0.00634	1.000
5	D5S424	93.23	3.14	0.00085	0.752
7	D7S502	80.86	2.55	0.00545	1.000
8	D8S585	47.96	2.62	0.00438	0.996
8	D8S531	63.93	2.45	0.00705	1.000
8	D8S281	119.66	2.80	0.00254	0.970
10	D10S196	71.95	3.15	0.00082	0.740
15	D15S1507	68.15	2.67	0.00375	0.992
15	D15S216	75.09	2.41	0.00796	1.000
17	D17S1795	77.3	3.27	0.00054	0.626
18	D18S469	106.38	3.00	0.00137	0.890
18	D18S1161	111.05	3.30	0.00048	0.592
20	D20S432	49.66	2.57	0.00504	1.000
21	D21S1898	38.62	2.71	0.00338	0.988

Singlepoint scores per marker.

**Table 3:** Multipoint linked regions with adjusted p-value<1.0.

Chromosome	Low(cM)	High(cM)	max(Z)	min(p)	Adjusted p-value
1	20	21	2.73	0.00315	1.00
1	167	171	2.86	0.00215	1.00
3	53	58	4.40	0.00001	0.20
4	130	131	2.40	0.00819	1.00
5	92	96	2.85	0.00217	1.00
7	81	81	2.38	0.00862	1.00
8	45	48	2.87	0.00207	1.00
8	64	65	2.43	0.00757	1.00
8	120	121	2.56	0.00530	1.00
10	72	72	2.99	0.00142	1.00
10	115	118	2.64	0.00421	1.00
15	65	68	2.75	0.00301	1.00
17	77	78	3.01	0.00133	1.00
18	82	83	2.42	0.00774	1.00
18	105	114	4.30	0.00001	0.25
20	50	50	2.40	0.00814	1.00
21	37	39	2.48	0.00658	1.00

**Figure 2:**
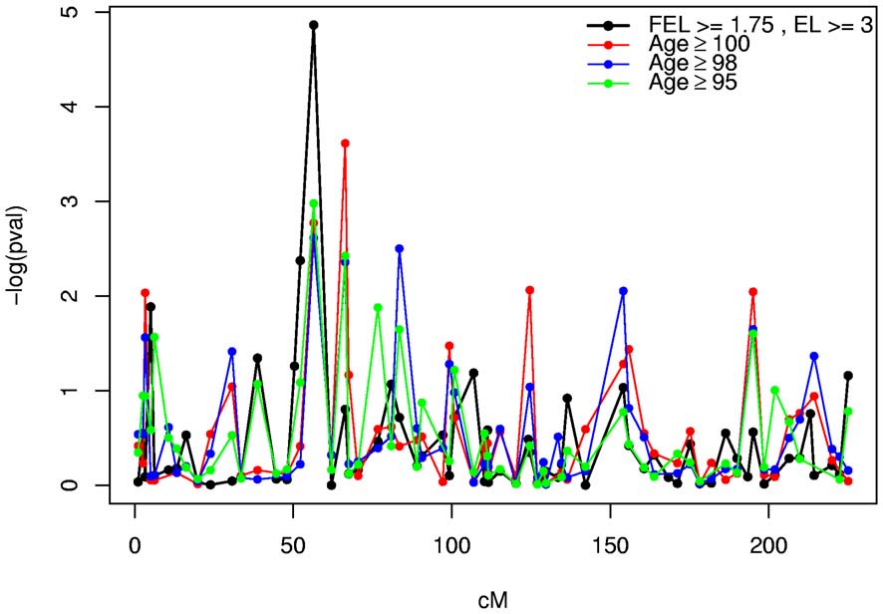
Comparison of singlepoint linkage results for chromosome 3 under varying definitions of affected status (nominal p-values).

To assess the degree of sensitivity of the linkage signal on chromosome 3 to the criteria we used to assign ELP “affection” status, we repeated the APM analysis and reassigned affected individuals 3 ways, depending on their attained age: 100, 98, or 95 years (regardless of sex or family history). Results are shown in Figure 2 where the highest peak corresponds to D3S3547 (56.45 cM), the same marker with the highest signal by the original APM test and definition of ELP affected. Shifting the criterion up for attained age and reassigning affection status accordingly, yielded peaks for the same markers, although the magnitude of each peak was considerably diminished. A similar reduction in evidence for linkage on 3p with increasing affection threshold was observed by Boyden and Kunkel [1]. The true centenarians (attained age of 100 yrs) exhibited a second peak at marker D3S3521 (64.35 cM), sufficiently close to the linked region reported here to warrant further investigation. It should be noted that increasing the attained age threshold for ELP substantially reduced the number of individuals counted as affected, especially among true centenarians (195 individuals, 67 related pairs aged ≥95; 88 individuals, 13 related pairs aged ≥98; 49 individuals, 9 related pairs aged ≥100). Therefore, the added linkage peak effect after adjustment may be due (all or in part) to an increase in noise as a result of a decrease in sample size.

## Discussion

**Figure 3:**
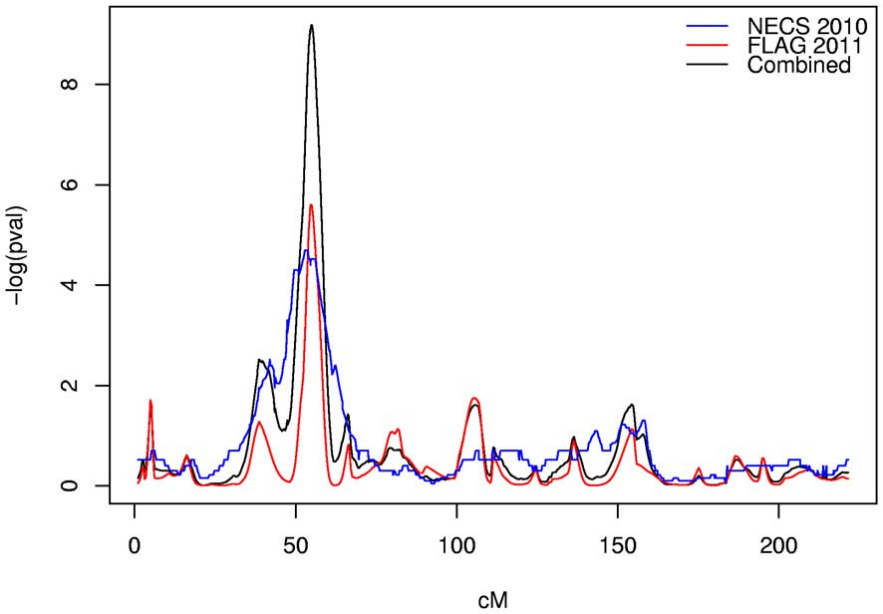
Comparison of chromosome 3 linkage results reported here to that reported by Boyden and Kunkel [**13**] (nominal p-values).

One immediate result of the FLAG study scan is an independent replication of Boyden and Kunkel's finding of a genetic linkage between excess longevity and a locus in the 3p22-24 region in the HMS data. Figure 3 compares the HMS and FLAG linked regions, based on Boyden and Kunkel's Age Category 4 (upper 2.5% of the 1900 birth cohort). Superficially, the HMS linked region appears broader than the linked region we have observed. This may be a consequence of design differences between the FLAG and HMS studies: a sib pair analysis led to the HMS result, while an analysis of a wide range of kin relationships led to the FLAG result, for approximately the same total number of pairs in each study. Our sample from UPDB's large multiplex families ensures greater variation in shared chromosomal segment length, and shorter average segments, among relative pairs contributing to linkage peaks. On the other hand, the microsatellite markers used in this study are less precise markers of location because the distance between them is much greater than for the SNP panel used by Boyden and Kunkel [1]. To remedy this, we are conducting fine mapping studies to more precisely define the shape and location of the linkage signal within the 3p22-24 region.

Figure 3 also shows results obtained by combining HMS and FLAG data, using the unweighted mean Z-score approach of Dempfle and Loesgen [35]. The combined data yield a linkage p-value of 1.005×10^−9^ (corresponding to an adjusted p = 0.008) at 55 cM on 3p. The combined data provide further support for Boyden and Kunkel's suggestive linkage peak at 9q31-34 for Age Category 8 (upper 0.5% of the 1900 birth cohort). We did not identify a peak in the same region from our data alone; however, the meta- minimum p-value by meta-analysis for the HMS 9q31-34 peak is 1.27×10^−5^ (adjusted p = 0.37) at 127.8 cM.

There are other suggested points of overlap between our results and the HMS study: their Age Category 10 (upper 0.2% of the 1900 birth cohort) suggests linkage to a region on chromosome 8q (meta p-value of 3.4×10^−4^ at 120 cM, corresponding to an adjusted p-value of 0.96). They found a similar suggestion of linkage to a region on 17q (meta- p-value of 7.3×10^−4^, adjusted p = 0.996, at 77 cM). However, our results do not indicate support for their chromosome 4 linkage peak, originally reported by Puca et al. [13], for their chromosome 12 peak reported in newly-enrolled HMS subjects, nor do the original HMS data show any indication of linkage anywhere on chromosome 18.

Human longevity is an outcome downstream of many biological processes, and it's very likely that multiple genes influence the trait. The FLAG study was designed to capture an extreme phenotype in a particular population, and our linkage results might be difficult to derive in other population settings. However, compared to most GWAS designs, the FLAG and HMS studies have better power to identify rare variants with large effects. While such variants may not explain a large portion of variation in longevity in a given population, they might very well help to identify important mechanisms that regulate onset and/or rates of aging in general. We should also consider that particular organs of the human anatomy might respond variably to mechanisms of aging, so that variation in aging among organs might suggest areas particularly amenable to pharmacological intervention.

Genes in the consensus linked region of 3p22-24 are listed in Table 4. The consensus region is the largest region for the combined data with a meta- p-value<0.001. Note that this definition narrows the region of interest more specifically to cytogenetic band 3p23-24.1. Of particular interest on 3p24.1 are *EOMES*, *CMC1*, and *AZI2* because of their potential interactions with mTOR/rapamycin [36], free radical production and detoxification [37], [38], and apoptosis [39], [40], respectively. Another gene of particular interest in the 3p region is *TGFBR2* because it is implicated in multiple disease etiologies [41], [42]), but of the 27 genes in the same region, most have functions that are not fully understood.

**Table 4:** Genes in 3p22-24 linked region.

Symbol	Start	Stop	Entrez	Name
NEK10	27232101	27385916	152110	NIMA (never in mitosis gene a)- related kinase 10
SLC4A7	27389218	27473249	9497	solute carrier family 4, sodium bicarbonate cotransporter, member 7
EOMES	27732890	27738789	8320	eomesodermin
CMC1	28258128	28336267	152100	COX assembly mitochondrial protein homolog (S. cerevisiae)
AZI2	28339090	28365579	64343	5-azacytidine induced 2
ZCWPW2	28406991	28541636	152098	zinc finger, CW type with PWWP domain 2
RBMS3	29297807	30026890	27303	RNA binding motif, single stranded interacting protein 3
TGFBR2	30622998	30710637	7048	transforming growth factor, beta receptor II (70/80 kDa)
GADL1	30742696	30911157	339896	glutamate decarboxylase-like 1

Start and stop positions are given relative to the Human March 2006 (NCBI36/hg18) assembly.

Our definition of familial longevity (elevated *FEL*) assumes a dominant or additive model of inheritance. As a consequence, our selection criteria for affected individuals (and their familial relationship networks) are less sensitive for identifying potential recessive traits than dominant or additive traits. The familial recurrence pattern expected for a recessive trait (sib pairs scattered throughout a pedigree), would in most cases result in higher than expected *FEL*, particularly in the large sibships typical of the UPDB. Nevertheless, our study has substantially less power to identify loci with recessive effects on longevity: only 63 affected sib pairs are informative with respect to recessively inherited factors; while 607 pairs are potentially informative for dominant or additive inheritance.

We have recently described patterns of gene expression that are associated with human longevity and aging [43]; now we can try to narrow the set of longevity-associated candidate variants at the 3p locus by searching for markers (microsatellites or SNPs) in the same 3p region that are associated with variation in gene expression patterns that are also associated with longevity. Although in principle this requires a complete set of GWAS data for each expression quantitative trait locus (or eQTL) of interest, several established data repositories for genome-wide eQTL studies [44], [45], should greatly simplify the process.

The FLAG study differs from the HMS and other studies of exceptional longevity in several important respects. We ascertained subjects on the basis of both familial longevity and personal longevity, which increases the probability that subjects carry a longevity-predisposing variant, but also increases the probability that any such variant is not widely distributed in the population. Our subjects were all of primarily Northern European ancestry, and hence were genetically less diverse than would be optimal for maximum generalizability of our results. We selected pairs of individuals related to varying degree, sometimes distantly related, which allowed us to identify a fairly large sample, but prevented us from using the most powerful techniques of linkage analysis. That we did not identify some linkage peaks previously reported by Boyden and Kunkel [1] or Puca, et al. [13] might be the result of relatively low power, allelic heterogeneity, and/or differences in study design. Our observation of a linkage peak on 18q that is clearly not present in the HMS data might similarly be the result of any of those factors. Given the substantial differences between the FLAG and HMS studies, and their limitations, it seems striking that the 3p23-24.1 region was clearly identified in both. Moreover, while our data do not quite replicate linkage peaks on 9q, 8q, and 17q, there is considerable support in both data sets for the possibility that predisposing variants are present in those regions as well.

It is likely that variants at many loci contribute to the heritability of longevity in humans. Our independent replication of the 3p22-24 linkage peak originally reported by Boyden and Kunkel [1] should help focus the search for variants associated with longevity in this relatively small region. Advances in DNA sequencing make it practical to rapidly sequence the exons in the region, or the entire region, and hence identify the variant(s) responsible for the observed linkage signal. Other regions identified in one or both of these studies also deserve further scrutiny.
